# Ansa Cervicalis Stimulation During Non–Rapid Eye Movement Sleep in a Patient with Obstructive Sleep Apnea

**DOI:** 10.1164/rccm.202411-2122IM

**Published:** 2025-06-02

**Authors:** Yike Li, Alan R. Schwartz, David Zealear, Megan E. Hall, Matthew S. Shotwell, Christopher J. Lindsell, Silvana Bellotto, Katherine E. Estes, Carol LeeAnn Wells, David T. Kent

**Affiliations:** ^1^ Department of Otolaryngology—Head and Neck Surgery and; ^2^ Department of Biostatistics, Vanderbilt University Medical Center, Nashville, Tennessee;; ^3^ Department of Otorhinolaryngology, University of Pennsylvania Perelman School of Medicine, Philadelphia, Pennsylvania;; ^4^ Universidad Peruana Cayetano Heredia School of Medicine, Lima, Peru; and; ^5^ Department of Biostatistics and Bioinformatics, Duke University School of Medicine, Durham, North Carolina

Hypoglossal nerve stimulation is a surgical treatment option for patients with obstructive sleep apnea (OSA) and positive airway pressure intolerance, but responses remain suboptimal (1, 2). Prior work demonstrated that caudal tracheal traction improves airway patency (3) and that infrahyoid muscle activation exerts caudal traction on the pharynx during ansa cervicalis stimulation (ACS). ACS increases retropalatal cross-sectional area and peak inspiratory airflow (V_I_max) and decreases measures of pharyngeal collapsibility in sedated patients (4, 5), but its effect on airway patency during natural sleep remains unknown.

**Figure 1.**
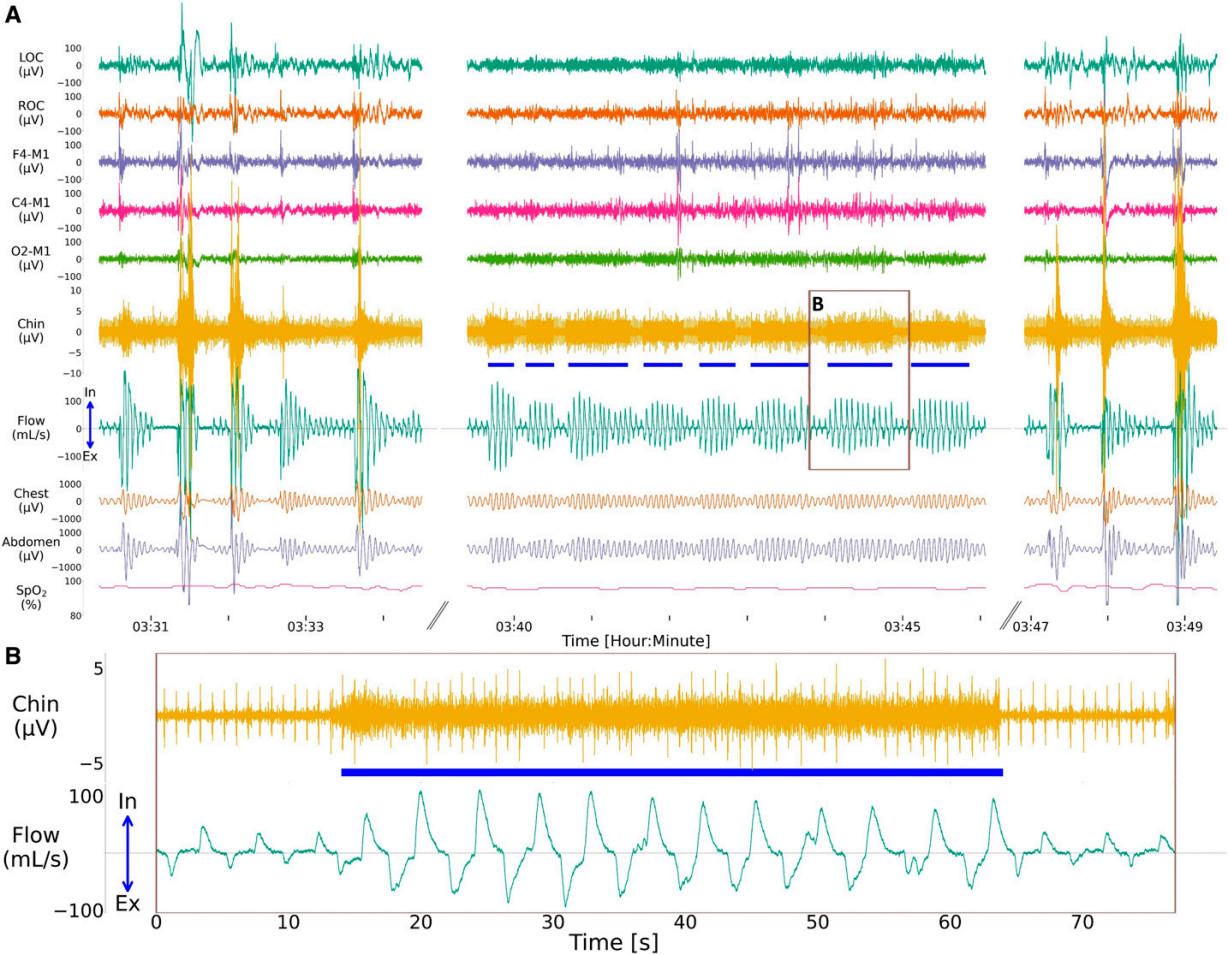
(*A*) Polysomnographic recording with (center) and without (left and right) ansa cervicalis stimulation (ACS) during non–rapid eye movement sleep. ACS was manually applied repetitively for 5–15 contiguous breaths (middle; blue dashed intervals mark stimulation artifact in EMG followed by several intervening breaths off ACS). Discontinuation of ACS caused an immediate return to baseline with markedly reduced tidal airflow comparable to that during unstimulated periods with recurrent obstructive hypopneas (right and left). (*B*) A single burst of ACS across 12 breaths generated EMG artifact and an associated increase in tidal airflow. “Chest” and “abdominal” indicate piezoelectrode gauges. Chin = chin electromyography; F4-M1, C4-M1, O2-M1 = frontal, central, and occipital electroencephalogram; Flow = pneumotachometer with open nasal mask; LOC = left electrooculogram; ROC = right electrooculogram; SpO_2_ = oxygen saturation as measured by pulse oximetry.

ACS was evaluated during non–rapid eye movement sleep (initiated with 10 mg zolpidem) in a 42-year-old man with severe OSA (apnea–hypopnea index, 56.1 events/h) and a body mass index of 26.0 kg/m^2^ who underwent bilateral ACS with hook-wire percutaneous electrodes during polysomnography. Without ACS, the patient exhibited obstructive hypopneas, leading to oxygen desaturations and arousals (Figure 1, left and right). ACS (amplitude, 1.8 mA; pulse width, 200 μs; frequency, 33 Hz) was intermittently applied for 5–15 contiguous breaths (Figure 1, center) using a manually toggled switch on the neurostimulation unit, resulting in immediate marked responses in V_I_max. Mean V_I_max significantly increased from 71.2 at baseline to 191.4 ml/s, representing an increase of 120.2 ml/s (95% confidence interval, 109.0–131.4). Ventilation was sufficient to prevent recurrent arousals from non–rapid eye movement sleep and stabilize oxygenation for 6–7 minutes. These findings suggest that ACS increases airway patency and is tolerable during sleep, supporting its viability as a potential OSA therapy.
